# Attachment and autism spectrum conditions: Exploring Mary Main’s coding notes

**DOI:** 10.1177/2516103218816707

**Published:** 2019-01-10

**Authors:** Barry Coughlan, Tess Marshall-Andon, Julie Anderson, Sophie Reijman, Robbie Duschinsky

**Affiliations:** 1 University of Cambridge, UK; 2 University of Kent, UK

**Keywords:** Ainsworth Strange Situation Procedure, attachment, autism, developmental psychology, differential diagnosis

## Abstract

Distinguishing autism spectrum behaviors from behaviors relating to disorganized attachment can be challenging. There is, for instance, a notable overlap between both conditions in terms of behaviors deemed stereotypical. In addition, there are also similarities regarding some atypical social overtures. Responding to this overlap has been the subject for much debate in the literature. Disorganized attachment was first introduced and conceptualized by the attachment researcher, Mary Main. Main is considered the leading authority on coding this phenomenon. During the course of archival research, we obtained Main’s notes on coding attachment in a group of 15 children with autism spectrum conditions (hereafter ASC). Drawing on these texts, this article explores Main’s reasoning when making distinctions between ASC and attachment at the behavioral level. Our approach is informed by Chang’s argument for the potential of “history as complementary science.” Analysis indicates that, for Main, frequency and timing were important differential factors when attributing a behavior to either ASC or the child’s attachment pattern.

## Introduction

Differential conceptualization is a routine activity for clinicians working with children and adolescents. Yet given the range of symptom manifestations, high levels of comorbid conditions, and shared symptomology between presentations, developing a clear clinical picture remains a challenge for clinicians today. One such area is the diagnosis of autism spectrum conditions (hereafter ASC), and urgency is added by the fact that the number of cases is increasing (Taylor, Jick, & MacLaughlin, 2013). It is becoming increasingly recognized that ASC shares phenotypic similarities with other traditionally distinct developmental conditions. Uncovering the differential features is consequential, given that access to supports, services, and the clinical and educational response is largely dependent on the clinical conceptualization of the case. A recent review by McKenzie and Dallos (2017) observed that research literature to date offers little practical guidance in respect to differentiating ASC from issues around child attachment, given that they have symptoms in common. Adding to clinical complexity, the two conditions may co-occur. Children with ASC do develop the entire range of attachment relationships (Rozga et al., 2018; Rutgers, Bakermans-Kranenburg, van Ijzendoorn, & van Berckelaer-Onnes, 2004; Teague, Gray, Tonge, & Newman, 2017), including disorganized attachment.

Here we report on an unusual source of information relevant to the differentiation of ASC and disorganized attachment. Mary Main is the researcher who first discovered and defined disorganized attachment, and she is regarded as the most authoritative coder of the phenomenon. In the course of archival research in her papers, detailed notes were discovered from her work coding a sample of children with ASC as part of a short-term longitudinal study (Rozga et al., 2018). The notes were only intended at the time for Main’s personal use; the remarkable extent to which they document what Main was perceiving and thinking is a reflection much more of Main’s unusual attention to detail than of usual coding practice. We received and are reporting on these notes with Main’s consent. These coding notes are of considerable historical interest as they offer a window into Main’s reasoning about how disorganized attachment and ASC might best be distinguished at the behavioral level. The coding notes cannot provide a model for making this distinction; for this, a better methodology would be to have teams of experts in both assessments code the same tapes and then discuss their conclusions. Our ambition here is not to develop such a model but instead to explore the reflections of the leading authority on disorganized attachment as she worked to code a sample of children with an ASC diagnosis. Despite these limitations in scope, such an exploration of the coding notes nonetheless raises issues for consideration regarding differential diagnosis.

## Literature review

### Assessment: ASC

Currently, ASC is considered a lifelong neurodevelopmental condition which, at its core, is characterized by atypical social communication and restricted and repetitive behavior across different contexts (American Psychiatric Association, 2013). Early clinical descriptions regarded autism as a rare discrete condition (e.g., Kanner, 1943). However, the latest edition of the *The Diagnostic and Statistical Manual of Mental Disorders* (5th ed.; *DSM-5*; American Psychiatric Association, 2013) marked a significant formal change in how diagnosticians conceptualize these features. That is, the nature of the variable, that is, autism, has changed from a categorical condition to a continuum, thus making it a spectrum. A key to this change has been the acknowledgment of the heterogeneity of symptom manifestations and diagnostic issues with comorbidity.

**Table 1.** Discriptive Statistics and Attachment Classifications.

Case ID	Sex	D autism	Primary classification	Alternative classification
31	M	D autism	D	C1
23	F	Not assigned	D	B2
20	M	Not assigned	B1	CC
26	M	D autism	D	CC
19	M	Not assigned	D	C
7	F	Unclear	D	B4/CC
34	F	D autism	B	D
41	F	D autism	B1	CC/C
5	M	D autism	B2	A2
29	M	Not assigned	B1	B3
28	F	D autism	D	B/A1
35	M	D autism	B3	Uncodable for D due to parental intervention*,**
37	M	D autism	A1	Not assigned
42	M	D autism	A2	A1

*Signifies that there was difficulty coding D in the child.

**Case 90 where the overall classification was unavailable.

In terms of social communication, the DSM offers several examples of features that are considered expressions of ASC. Difficulties with social reciprocal conversation, atypical eye contact, and difficulties with understanding social relationships are among some of the features described in the diagnostic manual. However, conditions characterized by atypical social communication are not uncommon in the DSM (e.g., social communication disorder and social anxiety disorder). Therefore, it is perhaps unsurprising that the other domain, ritualized and repetitive behaviors, is not only hallmark characteristics of ASC but also often differential when considering alternative socio-communication difficulties. Examples of restricted and repetitive behaviors include stereotypical movements, echolalia, atypical sensory interests, and emphasis on routine (American Psychiatric Association, 2013).

From an assessment perspective, clinicians rely on a range of standardized behavioral observations, interviews, and survey measures. While a range of these tools exists, it is widely acknowledged that a combination of the Autism Diagnostic Observation Schedule (ADOS-2; Lord et al., 2012) and Autism Diagnostic Interview Schedule (ADI-R; Rutter, Le Couteur, & Lord, 2003) constitutes “the gold standard” for assessing ASC (de Bildt et al., 2004; Le Couteur, Haden, Hammal, & McConachie, 2008; Rutter et al., 2003). While there is little doubt that these tools have played a vital role in helping to refine understanding of ASC, they are often unable to differentiate between ASC and symptomatically similar presentations such as issues around attachment (Woolgar & Scott, 2014).

### Assessment: Child attachment

The attachment system is a motivational system with the principle function of gaining access or proximity to the attachment figure (Bowlby, 1969). This biologically based behavioral system is activated when the child recognizes real or perceived threat or separation. Attachment behaviors are varied (e.g., crying, clinging, running) and change with development, but all serve the function of gaining access to the attachment figure. The majority of studies on childhood attachment use the Strange Situation Procedure (SSP) to classify attachment in infancy (Ainsworth, Blehar, Waters, & Wall, 1978). This laboratory-based procedure provides a snapshot of infant attachment related and exploratory behavior following two separations and reunions with their caregiver (Ainsworth et al., 1978). The following are the sequential episodes of the assessment:

1. The child and caregiver enter the room.
2. The child is afforded the opportunity to habituate to the room and explore/play with while the caregiver is present.
3. A “stranger” then enters the room and gradually seeks interaction with the child.
4. The caregiver leaves the room and the infant is left in the room with the “stranger.”
5. [Reunion 1] The caregiver returns to the room and the stranger leaves. At the end of this episode, the caregiver leaves.
6. The child is now alone in the room.
7. The stranger reenters the room and interacts as indicated by the child’s needs/signals.
8. The Caregiver returns [Reunion 2] and the stranger leaves.

The function of these different episodes is to offer a baseline and critical moments from which to consider behavior within the attachment-relevant contexts of separation from and reunion with a familiar caregiver.

Ainsworth, Blehar, Waters, & Wall (1978) observed that infants typically displayed one of three patterns: Secure (B), Avoidant (A), and Ambivalent or resistant (C). According to Ainsworth and colleagues, infants were considered Securely (B) attached if they used their caregiver as a secure base for exploration, showed signs of missing their parent during separation, and greeted their parent with an overt gesture and actively sought parent on reunion (Ainsworth et al., 1978; Solomon & George, 2008). Children who were classified as Avoidant (A) showed little sign of affect toward their caregiver upon entering the room, showed little distress during the separation, and actively avoided their caregiver during reunion. Despite inhibiting their reactions, Ainsworth et al. (1978) pointed to physiological evidence to suggest that the attachment system is still being activated in these children. Based on her observations of these infant–caregiver dyads at home, Ainsworth et al. proposed that their avoidant behavior was a response to common experiences of rebuff from their caregiver when distressed. Ambivalent–resistant children (C) on the other hand showed weak exploration of the room, showed overt signs of distress during separation, and combined displays of distress and anger and attempts to cling to the caregiver on reunion (Ainsworth et al., 1978). Based on her observations of these infant–caregiver dyads at home, Ainsworth et al. proposed that their ambivalent and resistant behavior was a means to maintain the attentiveness of a caregiver who, at home, tended to delay responding or whose availability might be inconsistent. Meta-analytic reviews have found that, except for samples of families under particular stress, cross-culturally, around 70% of children are classified as (B) Secure, 20% as Avoidant (A), and 10% as Ambivalent/resistant (Mesman, van IJzendoorn, & Sagi-Schwartz, 2016).

Yet through the 1980s, researchers began to note that some of the patterns of behavior the children were displaying did not align with those described in the original Ainsworth system. Following a systematic exploration of 200 SSP videotapes, Main and Solomon (1990) proposed a new “disorganized” (D) attachment classification. In their protocol for coding the new classification, they distinguished seven distinct indices (Main & Solomon, 1990):

- Sequential display of contradictory behavioral patterns;
- Simultaneous display of contradictory behavioral patterns;
- Undirected, incomplete, and interrupted movements and expressions;
- Stereotypies, asymmetrical movements, mistimed movements, and anomalous postures;
- Freezing, stilling, and slowed movements and expressions;
- Direct indices of apprehension regarding the parent;
- Direct indices of disorganization or disorientation.

Disorganized attachment behaviors are not necessarily pervasive and may only become apparent for brief moments during the SSP. As a result, children who receive a “disorganized” primary classification also receive, where possible, a secondary alternate “organized” classification. As guidance for coders in handling the issue of pervasiveness, Main and Solomon (1990) constructed a 9-point scale, with scores of more than 5 leading to a disorganized classification. In low-risk community samples, rates of disorganized attachment are around 15%; but this can increase to the majority of a sample among families known to social services for maltreatment, among families with traumatized parents, and among families facing multiple compounding socioeconomic risks. Main and colleagues theorized that the display of these behaviors, especially on reunion with the caregiver, understood to reflect a child’s experience of conflict or fear regarding that caregiver. Yet there can be many reasons for conflict or fear, as shown by the diversity of factors that increase its prevalence across different samples (Granqvist et al., 2017). It is also suspected that the behaviors may well also have a different meaning if they occur when a child is more alarmed or less alarmed than the degree evoked by the Strange Situation; as such, Granqvist and colleagues (2017) caution against coding disorganization using the behaviors specified by Main and Solomon outside the context of the SSP, as their validity for such application is presently unclear.

### Phenotypic similarities: ASC and attachment-related difficulties

One of the key features of ASC is “deficits in social reciprocity from abnormal social approach to reduced emotions or affect to failure to initiate or respond to social interactions” (DSM-5; American Psychiatric Association, 2013). This avoidance of social interactions appears similar to that characterized by the classification of thoroughgoing avoidance (A1) in the Ainsworth system, which describes failure to initiate or respond to social interactions with the caregiver on reunion. On this basis, it would be reasonable to expect that children with ASC would be overrepresented in the avoidant category. However, published studies indicate a lower prevalence of avoidant attachment in this group, with between 7% (Capps, Sigman, & Mundy, 1994) and 15% (Naber et al., 2007) of children with ASC classified as insecure-avoidant.

However, there are also a number of symptomatic similarities between the behaviors listed under indices IV of Main and Solomon’s classification scheme for disorganized attachment and the diagnostic criteria for ASC. For example, “rhythmical, repeated movements without visible function” is an indicator of disorganization (Main & Solomon, 1990), while “stereotyped or repetitive motor movements, use of objects, or speech (e.g., simple motor stereotypies, lining up toys or flipping objects, echolalia, idiosyncratic phrases)” are features of ASC behavior (American Psychiatric Association, 2013, p. 50; Lord et al., 2012; Rutter et al., 2003). Main and Solomon (1990) describe a case in which the infant would put her hands to her ears in response to being picked up by a caregiver (p. 143). In this case, the caregiver was found to be abusive. Interestingly, the ADOS-2, the direct behavioral observation used to diagnose children with ASC, gives a similar example in the “Stereotyped Behaviours and Restricted Interests’ section part D4. The instructions state “putting his or her hands over and/or fingers in his or her ears should be coded here” (ADOS-2, Module 3, Coding system, Lord et al., 2012). This specific example highlights an important difference between the coding of the SSP and the ADOS-2 and the need for informed clinical judgment. In the SSP, Main and Solomon (1990) are clear that stereotypy is coded as indicating disorganization when it occurs in contexts of relational stress such as reunion more than at pre-separation baseline and that it cannot be coded in the absence of the parent. In the ADOS, however, it remains unclear whether the observer would make this distinction when coding this behavior. On similar note, Main and Solomon (1990) propose that “asymmetries of facial expression directly upon the appearance of the parent” are indicative of disorganized attachment. Facial expressions are also deemed important in the diagnosis of ASC. For instance, Items 51, 57, and 58 of the ADI-R, the parental interviews used to assess and diagnose ASC, are concerned with the range and ability of the child to use facial expressions in the section on social development and play (Rutter et al., 2003). In this diagnostic interview, parents report on their experiences of the child’s social responses. It is likely that the asymmetries in social expression associated with disorganized attachment could receive a mark on Item 58 of the ADI-R, which describes “facial expressions slightly or occasionally inappropriate or odd” (ADI-R interview protocol: 52; (Rutter et al., 2003). Similarly, Main and Solomon (1990) observed asymmetrical facial expressions, but these were among children from maltreated samples on reunion with their caregiver in the SSP. Such overlap is potentially troubling from the point of view of differential diagnosis, even if both the ADI-R and the Main and Solomon protocols state that any such behavior must be considered in the context of other behaviors and features to warrant a classification. Good diagnostic practice always takes context seriously, but where behavioral markers overlap and the context for interpreting them may be ambiguous or difficult to discern, difficulties for clinicians will arise.

There have been calls to catalog the overlapping and distinguishing features of ASC versus attachment-related difficulties. Based on clinical observations, Moran (2010) developed the Coventry Grid to initiate a conversation around how to go about making this differentiation. The Grid outlines some of the overlapping domains of difficulties for children with both conditions (e.g., focus on routine, repetitive language, food sensitivity, attachment to preferred objects, atypical play, or atypical social interactions). It is not uncommon to hear references to the Grid in clinical practice, and there have been attempts to repurpose the Grid into a formal clinical interview (Coventry Grid Interview; Flackhill, James, Soppitt, & Milton, 2017). In a recent critical review of the literature, McKenzie and Dallos (2017) state that although the Grid does neatly describe the difficulties clinicians face when differentiating ASC from attachment-related difficulties, one problem with the Grid is that it combines the insecure attachment classifications: avoidance, resistance and disorganization, which represent quite a different phenomenon. There is also a paucity of research into the psychometric properties of the Grid, though the unpublished doctoral thesis of Kendall-Jones (2014) offers favorable indications regarding the internal consistency of the subscales as well as adequate face and content validity. Nevertheless, it is worth restating the original mission described by Moran (2010) which was to ignite a discussion regarding the difficulties in this complicated area of diagnostic practice.

### Assessment: Attachment in children with ASC

Adding to complexity for clinicians is the fact that not only may it be difficult to distinguish certain symptoms of ASC from disorganized attachment, but it is quite possible for the two phenomena to co-occur. Early commenters mistakenly presumed that ASC was incompatible with attachment (e.g., Bettelheim, 1967; Cohen, Paul & Volkmar, 1987; Kanner, 1943, 1960; Mahler & Furer, 1968; Rutter, 1978). However, this early hypothesis has been discredited by a host of studies (see Rutgers, Van Ijzendoorn, Bakermans-Kranenburg, & Swinkels, 2007). Researchers have demonstrated that children with a diagnosis of ASC show a range of attachment behaviors toward caregivers. Gestures, vocalizations, and preferential behaviors including proximity seeking for caregivers have been seen in children with ASC (Rogers, Ozonoff, & Maslin-Cole, 1993; Shapiro, Sherman, Calamari, & Koch, 1987), and many children with ASC are classified as securely attached by blind coders of the SSP. In the first study to apply the disorganized classification for a sample of children with ASC, Capps, Sigman, and Mundy (1994) looked at the attachment profiles of 19 children with autism using a modified SSP where the child in which the second separation was curtailed as the stranger did not leave the child alone in the room. Fifteen of the sample were deemed “classifiable” on the basis of the Ainsworth coding protocols, while the entire sample displayed evidence of attachment to caregiver. All 15 of the “classifiable” children had sufficient markers of disorganized attachment to be given this as their primary classification. Secondary classifications were secure (40%), insecure-avoidant (7%), insecure-ambivalent (13%), and insecure-undetermined (20%). Yet, in fact, only three of the children (20%) were considered to be truly disorganized when potential ASC traits were excluded from the analysis (Capps et al., 1994). Such findings agree with concerns among clinicians that ASC behaviors may be misidentified as behaviors related to disorganized attachment, and vice versa. Attempting to respond to this issue, Willemsen-Swinkels, Bakermans-Kranenburg, Buitelaar, Van Ijzendoorn, and Van Engeland (2000) distinguished among the Main and Solomon indices in a study of children with Pervasive Developmental Disorder (PDD). When Index IV behaviors (stereotypies) were removed from the analysis of disorganization/disorientation, only 16.5% of the children were classified as disorganized compared to 10.5% of normally developing children. Interestingly, even with Index IV removed, the prevalence of disorganization increased to 53.8% among children with both PDD and an intellectual disability (Willemsen-Swinkels, Bakermans-Kranenburg, Buitelaar, Van Ijzendoorn, & Van Engeland, 2000)

In a meta-analytic review, Rutgers, Bakermans-Kranenburg, van Ijzendoorn, and van Berckelaer-Onnes (2004) found that 53% of children with ASC develop secure attachments. In a more recent review, Teague, Gray, Tonge, and Newman (2017) found rates of secure attachment ranged from 40% to 63% with a mean rate of 47% (Teague et al., 2017) for children with ASC. Although this figure is lower than rates of secure attachment among children without ASC, it nevertheless makes clear that infants with ASC can and often do form secure attachment relationships. It is also worth noting that the same review found that the discrepancy between children with ASC and neurotypical children disappeared when controlling for cognitive ability or level of severity of ASC symptoms (Rutgers et al., 2004). Level of intellectual functioning and severity of ASC symptoms is recurrently linked to lower attachment security (e.g., Naber et al., 2007; Rogers & DiLalla, 1990). In addition to the lower rates of attachment security, the review by Teague and colleagues (2017) also found that children with ASC had higher rates of disorganized attachment: approximately 22% compared with 15% in low-risk neurotypical samples. Most recently, Rozga et al. (2018) note that the theoretically expectable link between parental sensitivity and infant security is present in samples of children with ASC with relatively fewer cases coded as disorganized (e.g., Oppenheim, Koren-Karie, Dolev, & Yirmiya, 2012), whereas in samples with ASC where most children are also coded as disorganized (e.g., Van IJzendoorn et al., 2007), there is no relationship between parental sensitivity and the child’s attachment classification.

McKenzie and Dallos (2017) have challenged the prevalence literature, arguing that “autism and attachment difficulties result in similar symptoms and even very experienced clinicians find identifying which symptoms are attributable to autism extremely challenging” (p. 636). Merely cutting out Index IV behaviors, the strategy pursued by Willemsen-Swinkels and colleagues is regarded by McKenzie and Dallos as insufficient to adequately address the issue, though in our view this remains an open, empirical question. Nonetheless, we agree with McKenzie and Dallos that further efforts would be valuable to untangle the behavioral properties of ASC from disorganized attachment, even if absolute distinctions at an individual level will likely always retain some uncertainty. Although by no means able to offer a model for making such distinctions, this article will nonetheless attempt to speak to these concerns, making use of archival materials to explore strategies employed by Mary Main, the leading authority on coding disorganized attachment. In this, our approach is aligned with Chang’s (2017) argument for the potential of “history as complementary science” on the basis of three kinds of benefits that may be gained from history for science:

- increasing scientists’ critical awareness of the contingency of the present and expanding their understanding of the concepts they depend upon (what Chang terms “extension”);
- recovery of relevant ideas and aspects of methodology that have been lost beneath the headline stories of published results and conclusions;
- use of awareness of contingency, and recovery of ideas, to develop new hypotheses for testing.

## Method

In the course of archival research in Main’s papers, notes were obtained for 15 SSPs conducted on a sample of children with ASC who visited the UCLA Medical Centre between 1997 and 2000. These 15 cases represent 37.5% of the cases seen during this total period; the other notes were not found in the archive. All children in this study had had their diagnoses of ASC confirmed by both the Autism Diagnostic Interview–Revised (ADI-R; Lord, Rutter, & Le Couteur, 1994) and the Autism Diagnostic Observation Schedule–Generic (ADOS-G; Lord et al., 2000) methods. The children in the original study had a mean chronological age of 47 months and a mean language age of 21 months [Rozga et al., 2018]. The range of ages in the subset of notes available is unknown.

The notes are Mary Main’s own careful description and analyses of SSP recordings. They include detailed information about the child’s and caregiver’s behavior in each episode of the procedure. Within these notes, Main indicates which behaviors she would consider related to ASC (what she refers to in the notes as “Autistic D”), which she would consider signifiers of the attachment relationship (what she refers to as “true D”), and which might be related to both. These notes also include Main’s overall classification of the child’s attachment, with the exception of Case 30 which is missing Episode 8 and Main’s overall classification. We conducted a qualitative analysis of the notes, with three researchers reading each case multiple times, seeking to identify what behaviors prompted Main to make her assignation as well as the contexts in which these behaviors occurred. The conclusions of the researchers were then conferenced, in generating themes of particular focus to report. As a check on our interpretation of the notes, our findings were reported to two developmental scientists trained in coding the SSP, who provided helpful feedback and input based on their practical experiences as coders (Reijman, Foster, & Duschinsky, 2018).

## Results

### Stereotypies and repetitive behaviors: Context and frequency

In line with the written Main and Solomon (1990) protocols, Main consistently treated the context and frequency of stereotypies and repetitive behaviors as important and differential. Specifically, behaviors that appeared throughout the SSP and not exclusively during key moments—such as reunion—were typically considered features of ASC by Main rather than related to attachment relationship. This is evident in Case 41 where, during Episode 2, the child shows atypical vocalizations and has her “arms extended” and moves her wrists in a way that is described by Main as “autistic.” Then during Episode 4, when the mother leaves, the child expresses some atypical vocalizations and complex hand mannerisms. Main likewise does not consider these stereotypies a signifier of “true disorganization.” During Episode 8, however, Main observes that the child engages in hair twisting upon the entrance of her mother. Since this is the first instance of this behavior, and it occurs directly on reunion with the caregiver, Main considers this to be indicative of disorganization. Overall, this child was classified as autistic and securely attached (B) with their caregiver, despite displaying sufficient behavior from the indices of disorganized attachment that she would otherwise have likely received this classification.

An example of frequency playing a differential role can be seen in Case 35. In the early, pre-separation episodes, which are treated as a “baseline” for understanding a child’s ordinary behavior, the child engaged in “hand flapping” and atypical movements. Main reflects on this continuity between behavior pre- and post-separation in her remarks on Episode 5 where she observes the child “goes to the door, grinning, jumping, and hand flapping.” Main comments “we have seen this too often to call it regular D, it seems often to simply be his style of movement.” The behavior does not seem differentially related to key attachment stimuli such as separation and reunion, giving Main confidence that the behavior is solely expressive of ASC and not indicative of disorganized attachment. Again, the child was classified as autistic and as having a secure attachment with their caregiver.

Case 34 is much less clear-cut for Main. Here the child displays atypical movements and “stereotypy” in Episodes 2 and 3. During Episode 5, Main notes that as the caregiver enters, the child displays “many gestures of autism/excitement, legs spread, arms shaking as M enters, but these seem to be looks of pleasure.” Main muses to herself: “This is almost impossible to code.” She continues,

it would definitely be called D in the system for low risk samples, especially with throwing her head back, but on the other hand she just seems overwhelmed by excitement/ pleasure and unable to remain contained in her body. So, we code D = 5.

In the Main and Solomon (1990) coding system, a score of 5 is neither organized nor disorganized—it is the threshold between the two, and the coder has to make a qualitative decision about which side of the line to place the case. In making a D = 5 code, Main is therefore here expressing doubt as to whether the SSP with this child, without further understanding of the child’s behavior at home, conveys sufficient information to show what is going on. In her comments, Main reflects that these are “definite D gestures which may just be overexcited happiness.” Ultimately, Main concludes that the frequency of atypical movements and stereotypies across episodes is suggestive of ASC rather than disorganization. But she appears to suspect the key attachment situation of reunion is causing attachment processes and ASC to intersect, producing these behaviors suggestive of overwhelming excitement. She assigns the case a classification of “D autism” with an alternative secure (B) classification. But she notes to herself that if she is wrong in attributing the behavior to ASC, the next best account of the child’s responses in the Strange Situation would be disorganized attachment.

### Prone postures

The coding notes suggest that falling prone or lying to the side was a feature which was highly prevalent in this sample in the Strange Situation. In all cases, Main gave cases where a child fell prone a “True D” score—though the score it received depended on the specific posture. It appeared that if the child was fully prone, this led to the assignment of a greater “D score” on the 1–9 scale than if the child was observed lying on their side or leaning.

Movement while assuming the posture appeared to be of importance to Main; her published discussions suggest that she regards falling prone as a kind of tonic immobility in the context of dissociation (Main & Morgan, 1996), and so a child showing movement whist prone would reduce confidence that it was a dissociated state. For instance, in Case 19, during Episode 3, the child “lies on the sofa and bangs the orange toy and then lies still in an odd posture, but some movement, on stomach, slowly moving the toy.” For this episode of abnormal posture, he was given a “D score” of 3. This is lower than his “D score” of 6 in Episode 4 when “he moves around prone the other way, depressed posture, stilling, some slight hand movement.” Stilling, an Index V indicator of disorganization, appeared to be closely related to falling prone in this sample. However, since stilling by definition entails a lack of movement, in line with the Main and Solomon (1990) protocols that specify duration as an important criterion, a major additional factor used by Main in determining the “D score” for stilling was the length of time the posture was adopted. A good example of this is Case 28, Episode 7 where Main observes: “He then moves to M’s empty chair and pulls his whole body onto it, face turned in, anomalous and yet deliberate, depressed and waiting.” This is given a “D score” of 9. Main goes on to reflect that “we have never seen a child manage to do this before.” As this is scored during Episode 7, this did not contribute to the child’s overall score.

In Case 7, Main describes another child, whose attachment is difficult to classify. In this case, the child demonstrates some atypical gestures and posture in Episode 2. Episode 4 is characterized by “crying” and aggressive play with the toys. On the first reunion, the child “wipes hand across her eyes” and repeats this behavior when the caregiver moves closer. The child repeats this behavior in Episodes 6 and 7. During the final reunion in Episode 8, the child is observed to “move away” from the caregiver, which Main states “seems like a momentary sequential display of contradictory behaviors.” This is followed by the child wiping her hand across her eyes again. In the comments section, Main remarks “we are not familiar enough with autism to know whether the child’s tantrum and movements are characteristic” of ASC. However, she states that the behaviors in Episode 5 make an “organized” classification “difficult to assign.” This appears to be because the stereotypies intensify on reunion, suggesting relatively decisively that the behavior indicates tension or conflict about the relationship, especially since in Episode 8 the behavior is followed by direct conflict behavior regarding proximity with the caregiver.

In coding prone behavior, Main also observes that the response by the caregiver can make this uncodable. Potential disorganization of the attachment system can be camouflaged by parental intervention. This was noted in Case 35 during Episode 8 where the child briefly collapses to the floor in a depressed position. Six seconds later, the mother intervenes by correcting the child, sitting her upright. Main comments that the mother might be “breaking up the real D behaviour.” This intervention was consequential as Main concludes that, as a result, the procedure was “uncodable for real D.” The SSP requires that the parent’s behavior is sufficiently standardized that attachment behavior shown by the child toward the parent can be displayed without interruption.

### Aggressive behavior

Aggressive behavior toward the caregiver was mentioned by Main in several of cases. In the written coding system, aggressive behavior toward the caregiver while the child is apparently in a “good mood” is mentioned (Main & Solomon, 1990, p. 136). The coding notes give the impression that the criterion requiring “good mood” may have been relaxed, as aggressive behavior toward the caregiver without apparent good mood was nonetheless used as an indicator of disorganized attachment when the behavior was coupled with other indices of disorganization such as atypical postures and fear smiling.

In Case 23, there is “quasi-aggressive behaviour” with shrieking, kicking of legs and throwing of pillows, as well as some “clasping of her left wrist with her right hand.” The antecedents for this behavior are unclear as the caregiver and child are playing a game. In Case 31, the aggression is clearer. Main states that during reunion

he stares down at her aggressively and seems to hit at her with the cup. She says ow and he flails the cup down at her again but doesn’t hit her face. She’s restraining him whilst he flails the cup at her aggressively.

In these cases, Main codes the behavior as evidence of disorganization and these behaviors acquire particularly high “True D” scores of D = 5 and D = 6, respectively. It is worth noting that even Main does not seem to be sure of what is precisely going on in the final moments of Case 23 as she states “nothing in this final minute is clear except that C is excited, perhaps aggressive, and becoming disorganized.” Main is able to make the assignation of disorganized attachment without being sure exactly what is going on, since one factor used to weight cases toward a disorganized attachment classification to a case is when a coder sees behaviors listed by Main and Solomon but cannot figure out the meaning of their sequencing, since this implies a disruption of the effective functioning of the attachment system. Overall, Main seems to regard the potentially aggressive behavior as sufficiently contrary to the functioning of the attachment system that it indicates that this system is becoming disorganized. Although ambivalent-resistant children are also anticipated to show anger according to the Ainsworth coding protocols, Main seems to distinguish this display as disorganization because of the sharpness of the jerking away and the absence of any subsequent attempt to cling.

Previous research has established that atypical sensory interests or difficulties are a prevalent feature of ASC (e.g., Ben-Sasson et al., 2009). Sensory sensitivity, rather than sensory seeking, has been associated with aggressive behavior in this population (e.g., Mazurek, Kanne, & Wodka, 2013). In Case 31, as the mother picks the child up for a hug, “he seems to raise up to flail away, multiple jerks away from her” and then shows aggressive behavior. This is given a “True D” score of 5. Main states that this is indicative of true disorganization since the aggression seems to arise out of nowhere. Without having seen the tapes, it is impossible to appraise Main’s assessment of the particular case. It could well be that there appeared little relationship between the caregiver’s behavior and the child’s. However, we are struck that items regarding negative/aggressive responses to touch feature in a number of ASC assessment tools (e.g., The Diagnostic Interview for Social and Communication Disorders; DISCO; Wing, Leekam, Libby, Gould, & Larcombe, 2002). Therefore, it is possible that the same behavior, seen by a DISCO coder, would have been assigned as a marker of ASC rather than related to child attachment. In her coding notes, Main herself acknowledges that, without specialist training in assessment of ASC, she runs up against the limits of her expertise in such attempts to judge whether aggressive behavior toward the caregiver should be regarded as regarded as signally disorganization, ASC, or—potentially—both.

### Atypical facial expressions

From the coding notes, atypical facial expressions appear to have been prevalent in this sample. Commonly, these unusual facial expressions were described by Main as either “aggressive smiles” or “fear smiles.” These were not typically thought by Main to be autistic in nature and were instead either uncoded or coded as “True D.” A contributing factor was evidently that there was an abundance of these abnormal facial expressions directly on reunion, which suggested to Main that they offered a window into the quality of the attachment history with that caregiver. For instance, Case 26 displayed repeated and sustained atypical facial expressions. In Episode 5, it is noted that C “gets a very odd facial expression, makes anomalous almost inhuman noises with fear-aggressive grin at the wall. This goes on and on.” This extract leads to a very high score of D = 9. In Episode 5, the child’s odd facial expressions continue but seem to coincide with an element of contradictory behavior, which adds to the coder’s belief that the behaviors reflect disruption of the attachment system. “He moves away from, then approaches M with the same strange and stretched fear/aggression tooth-filled grin he had when distressed, putting his arms up to be hugged, she bends to hug him, his face is extremely odd.” This extract again scores highly with a score of D = 6.

The cause of the atypical facial expressions in this sample is explored by Main in her reflections on Case 41. This case is unusual in that the father happens to enter at the end of the normal SSP, which up to that point had involved the mother. It is also an anomalous case in that the child’s facial expressions have not been classified as disorganization, the implication being that they are instead due to ASC. In Episode 8, on reunion, Main’s notes state “as Mother leans towards her, talking, pleased smile, but then it takes on a strange element.” The notes report that “Mother caresses her leg, Child says ‘no’, Child’s face twists, very peculiar expression. The increase in odd facial expressions on touch indicates a possible autistic response.” However, Main does states that “there are a lot of very odd faces in this episode, which were rare before” which raises the possibility of disorganization since the context suggests that the behaviors are not pervasive, but specific to attachment-relevant interactions with the caregiver. It is not clear. (Although outside of the SSP and therefore technically uncodable, when the Father enters at the end, Main notes that the child has a “broad smile with a fear element.” In this reunion with father, Main wonders whether the conflict between approach and avoidance producing the smile with a fear element is caused by autism: It could be that autism is causing the child to be worried by proximity with the caregiver, producing phenotypic resemblance to disorganized attachment.)

If so then ASC here would be, technically, causing disorganization of the attachment system. But this may or may not serve as a marker of the history of the child’s attachment relationships or have the predictive significance associated with disorganized attachment in other samples, upon which its validity as a classification is regarded to rest by developmental scientists. The child may not necessarily be afraid or worried by the caregiver, but instead worried by the unwanted proximity they anticipate will be forced on them as part of expected child–caregiver routine. Indeed, Main suspects that some parents may have “pre-trained” their child to act affectionately on reunion, when their inclination—influenced or not influenced by ASC—might have otherwise not inclined them to do so. This can be seen in Main’s observations on Case 5. Main notes that when the mother and child exchange saying “I love you,” they do so in voices that “suggest she says this a lot, or it is part of a routine.” In her summary of this case, Main says “we again have the problem of some training in affectionate behavior,” indicating this is not an isolated case in this sample (though it is also likely not an isolated case in general as typically developing infants may well also be taught artificial or routinized sharing of affection).

## Discussion

McKenzie and Dallos (2017) have observed that even the most experienced clinicians find ASC and disorganized attachment difficult to differentiate. The current article sought to identify and examine some of the practical strategies used by Mary Main, the most authoritative coder of disorganized attachment, when classifying and reclassifying attachment in children with ASC. It is not our aim to evaluate Main’s coding but to understand the principles she’s using. Examination of her coding notes therefore offered special opportunity to see her reasoning regarding whether a behavior suggested this phenomenon, using history as “complementary” to science (Chang, 2017) in exploring ideas and aspects of methodology beneath the headline stories from published papers of results and conclusions.

Overall, it seems that Main has developed a number of strategies for considering what was attachment-related and what was autism-related. For Main, the clearest differential seemed to be the nature of the behavior. Stereotypies have become an established diagnostic feature of ASC while also being associated with disorganized attachment (American Psychiatric Association, 2013; Main & Solomon, 1990). The majority of the children in this sample displayed stereotypies at some point in the procedure. Main’s coding notes highlight the value of considering the context in which such behaviors are occurring in order to evaluate their function and meaning. In general, Main considered stereotypies suggestive of disorganization only when they became apparent at moments that are understood to reflect the history of the attachment relationship—above all, reunion. Where they were more equally spread across episodes, they were attributed to ASC and deemed part of the child’s behavioral repertoire. Moreover, the form and sequencing of the behavior was also of concern to Main. Hand and arm flapping, toe-walking, and full-body circular motions were regarded as features of ASC. By contrast, jerky full-body movements away from caregiver and atypical movements with hands toward face or head were more frequently considered indicative of disorganization, perhaps given timing and context and also as these were more suggestive of fear or another aversive affect toward the caregiver.

Main also viewed falling prone a critical item for classification in this sample. Main and Solomon’s disorganization/disorientated classification scheme (1990) specifies under Index IV: “assumption of huddled, prone, depressed posture for more than 20 seconds, unless infant is clearly tired” indicates the presence of disorganization (Main & Solomon, 1990). This prone or depressed posture was seen by Main in 11 of the 15 children. Some children adopted a complex huddled posture; some simply lay on their stomachs; and others adopted what Main considered more submissive, limp postures or depressed leaning as opposed to the typical prone posture. Overall, it appeared that the less movement that occurs when the child is prone, the greater “D score” given by Main. Stilling, an Index V indicator of disorganization, appeared to be closely related. In line with the Main and Solomon (1990) written coding protocol, an additional factor in determining the “D score” for stilling appeared to be the length of time the stilling continued. The lack of movement in the prone postures, and the length of time in the stilled behavior, may have been given weight as markers of dissociation (Main & Morgan, 1996). It is important to note that the “D scores” obtained in some of these cases of falling prone or stilling were high enough in isolation to result in an overall D classification. Unlike stereotypies, atypical postures are not, technically, a diagnostic feature of ASC. However, there is evidence to suggest that children with ASC are at greater risk of delayed postural development in both sitting and standing (Nickel, Thatcher, Keller, Wozniak, & Iverson, 2013), although an alternative hypothesis would naturally be that higher rates of disorganization among children with ASC could contribute to the elevated rates of apparent postural developmental delay. Concerns regarding motor development and gait are long-standing in ASC research (see Rinehart et al., 2006). Given that falling prone is an unusual form of disorganization, but was common in this sample, future studies on attachment and ASC should consider the significance of atypical postures in children with ASC.

Aggressive behavior directed toward the caregiver was typically viewed by Main as a result of true disorganization, especially when coupled with other features of disorganization such as atypical postures and fear smiling. However, children with ASC typically experience more episodes of challenging behavior than atypical peers (Emerson, 1995; Holden & Gitlesen, 2006). It could well be that the aggression was potentiated by sensitivity to touch or as a function of communication. In coding a case of aggressive behavior, Main herself acknowledges that it would require greater knowledge of ASC than she possesses to feel confident in knowing the meaning of the particular behavior, and additionally such assessment may benefit from knowledge of the child’s behavior at home in order to give more context for understanding the meaning of behavior.

Atypical facial expressions were also prevalent in this sample. Main generally treated them as markers of disorganized attachment when the atypical expression included apparent anger or fear, and when it occurred at critical moments such as reunion. This is an important consideration for clinicians as it underscores the necessity of being context-aware when conducting diagnostic assessments. It also raises the question of what weight should be given to the affects seen displayed toward the caregiver, since both fearful and aggressive smiles seemed more common in this sample than would otherwise be expected. Main appears to wonder whether a quality in how some parents train children with ASC may cause such expressions, such as worry about expected physical contact. Main’s notes also suggest that some atypical facial expressions may reflect both ASC and disruption of the attachment system, though it is not clear whether such behaviors reflect the same process as usually ascribed to disorganized attachment or would have similar sequalae.

Our examination of Main’s coding notes throw into relief one of the major structural strengths of the SSP: It provides practitioners with a baseline and critical moments from which to consider behavior. Episodes and timing provide the practitioner and coder with context for the behaviors. This enabled Main to consider the context and frequency in her explanation of the features. It is a significant matter for further thought and discussion regarding whether the same considerations might be brought to bear while coding the ADOS. For instance, if an approach by a parent preceded the child putting their hands over their ears, would that specific instance be coded in the ADOS as suggestive of ASC? Moreover, the ADI-R is based on one parent’s experiences of the child: Behaviors that occur infrequently and only in the context of a particular infant parent–relationship could therefore be potentially coded as pervasive.

Yet, it is important to note that ASC and disorganized attachment operate in different discourses. ASC is a clinical diagnosis and the product of psychiatric discourse, whereas disorganized attachment is situated firmly within the realm of psychological discourse. Therefore, there may be conceptual or indeed epistemological tensions when trying to disentangle these conditions at a behavioral level. Moreover, it is not clear that the SSP will assist clinicians when thinking about differential conceptualizations.

## Conclusion

It is becoming increasingly acknowledged that differentiating ASC from attachment-related difficulties poses a challenge, in certain instances, for practitioners. We concur with Woolgar and Scott (2014) that clinicians must apply attachment theory to their case formulations with care, where ASC has been diagnosed or suspected, given the state of knowledge in this area. We anticipate that over the coming years, calls from clinicians for help in distinguishing ASC and attachment issues will be answered through the application of new approaches such as the network analysis of symptoms (Borsboom & Cramer, 2013) and attention to the role of general psychopathology as well as discrete diagnoses (Caspi et al., 2014). However, there are methodological and conceptual questions to answer as well, which cannot be solved by these new approaches alone. And for such questions, as Chang (2017) has argued, attention to the history of psychology offers a further source of light, in this case, permitting attention to how the concept of disorganization is used within coding practice; methodological challenges in using the concept; and generating potentially testable hypotheses regarding behaviors of potential relevance for making clinical distinctions.
